# Enhancing Teacher–Student Interaction and Students' Engagement in a Flipped Translation Classroom

**DOI:** 10.3389/fpsyg.2021.764370

**Published:** 2021-10-05

**Authors:** Yi Wei

**Affiliations:** Foreign Languages Department, Shijiazhuang Tiedao University, Shijiazhuang, China

**Keywords:** innovative approaches, flipped translation classroom, students' engagement, teacher–student interaction, translation research

## Abstract

Learning faculties are looking for innovative approaches to effective teaching in the translation process which can not only enhance students' engagement but increase the interactions between teacher and learners as well. With the amplified accessibility of network-centered instructive knowledge, teaching translation from the viewpoint of computer-aided instructions and online platforms have flourished. Flipped classroom (FC) is one of these new inclinations used in higher education nowadays which can attract stakeholders' attention. This review aims at exploring its effects on students' engagement and teacher–student interaction in translation classes. Some implications and suggestions have been presented for language teaching stakeholders in translation research.

## Introduction

There are currently remarkable issues about the translation courses, according to the surveys done in universities and colleges (Gong and Du, 2019). Students regularly deal with some problems including low achievement, lack of enthusiasm, engagement, and self-confidence (Joyce, 2018; Sun and Yuan, 2018). Significantly, translation instruction essentially follows the conventional mode, involving excessive education, insufficient class hours, inability to internalize translation theory, difficulty in shaping abilities, and inability to exercise translation thinking (Gong and Du, 2019). Since it is affected by the conventional educational model, the English translation course needs new educational ideas and approaches since their absence has truly blocked its improvement. Thus, changing the educational method of translation is a significant point, and it turned into a recent trend with the improvement of network innovation (Liu and Zhou, 2019). Indeed, being affected by conventional teaching, the progress of translation is hindered, so innovative teaching approaches in English translation courses should be accentuated (Wenwen and Yuying, 2020).

There are some different ways to assist learners to overcome these difficulties, one of which is to apply a learning method that is proper to solve the problems that learners generally come across in translation classes (Mannahali and Rijal, 2020). A method that has newly become popular in education is flipped classroom (FC) (Akcayir and Akcayir, 2018). In FC, teachers give learners asynchronous video speeches as assignments, thereby creating more opportunities for intuitive exercises in the class (Jensen et al., 2018). In addition, Bergmann and Sams (2012) have depicted the FC approach in a more extensive viewpoint, and they maintain that the FC approach is a setting where students take charge of their own education which *per se* builds connections among learners and educators. In FC, students' responsibility for the content is promoted inasmuch as the fact assignments are utilized in a way to help learners to find thoughts and ideas on their own (Strayer et al., 2016).

Likewise, exploratory tasks and class negotiations often happen in cooperative groups that consequently stimulating constructive peer-to-peer communications and augment teacher–student interpersonal communications (Xie and Derakhshan, 2021) that is considered as vehicles for increasing student engagement (Derakhshan, 2021). The positive relation between teacher and student is a facilitator of an extensive kind of proper learner-related consequences including engagement, success, and enthusiasm (Derakhshan et al., 2019; Pishghadam et al., 2021).

Among various factors, one justification for the interest in the FC could be the significant role of learners' engagement (Zainuddin and Halili, 2016) that has been at the center of attention lately due to the arrival of positive psychology (Wang et al., 2021). Student engagement is considered as a causative issue in education nowadays that refers to the strength and energy that students employ in the progress of their learning and it is manifested through the ranges of behavioral, cognitive, or affective criteria (Li, 2021). To preserve student engagement, another effective factor is the mutual relation between teacher and student (Pianta et al., 2012).

Indeed, FC has shifted the rights of learning from teachers to students (Sohrabi and Iraj, 2016) that cultivate autonomy. Learners learn via active learning practices that enhance their engagement and increase their critical thinking (Baepler et al., 2014). A major contribution of flipped education for many educators is the chance to involve learners in various learning activities in an environment where they, as well as the learners' classmates, are available to assist and cooperate (Hodgson et al., 2017). It is also important to note that changes have also occurred in the responsibilities and duties of learners; knowledge is no longer received passively by learners. Instead, they become active participants in classroom discussions through educators' guidance (Yanhui and Yi, 2014).

In the classroom, a major shortcoming of the translation classes is that learners do not receive the educators' prompt attention during the initial stages of absorbing and internalizing knowledge, which can act as an obstacle in this process (Yu, 2017). As the FC concurs with the hypothesis of constructivism (Bada and Olusegun, 2015), this issue can be settled in which translation education becomes like the fundamental scholarly translation knowledge, and translation procedures can be introduced before the class time to save time for learners to take on information through individual questions and shared learning, and subsequently study and merge information they have learned through the classroom communication (Yukun and Aili, 2014). The goal of a flipped translation education is unique in comparison to a conventional translation class where most of the class time is allotted to addressing translation procedures and working on translation assignments. The objective of FC for learners is to foster autonomy in learning translation and to utilize higher-order thinking to expand their translation ability (Lin, 2019).

On the one hand, the impact of the FC on learners' engagement and academic success are scrutinized in prior studies (Jamaludin and Osman, 2014; Jensen et al., 2018), and on the other hand, peer-peer and learner-educator communication were investigated in some other studies (McLean et al., 2016); however, to date, no FC researches had been carried out relating the above-mentioned issues in translation classroom. Therefore, this review aims to address the aforementioned gap, by considering the same issue in the translation classroom.

## Flipped Classrooms and Its Advantages

FC idea allows learners to study the material in advance, so teachers devoted class time for collaborative discussions (Rotellar and Cain, 2016). There have been many perspectives and observations about the success of FC instruction, as its popularity grows every year (Hall and DuFrene, 2016). One of its advantages is an increase in the efficiency of time management as FC usually involves watching a video version of the course material online as an assignment. So, students have the opportunity to be actively engaged in that material during class the following day (Martin and Rimm-Kaufman, 2015). Likewise, it permits educators to invest more energy in communicating with learners, which sets out more open doors to check for comprehension and clear up misguided judgments (Bergmann and Sams, 2012). Learners acquire information at home in a flipped approach through watching video slides prepared by the educator and practicing the abilities in class, where the educator can definitely regulate and manage the student (Chen Hsieh et al., 2017). Undoubtedly, as declared by Hung (2015), the learners' arrangement before the class is vital for them to have the option to be more included and to accomplish additional satisfying results. The principle of flipped learning is to involve learners through active learning conditions intended to prepare and rouse them to carry out appraisal assignments because of feedback presented via interaction through all the phases of learning.

The flipped approach intends to establish a learner-focused learning climate in which learners deal with their own learning and become more dynamic and intuitive in class which stimulates higher-order thinking, and improves educator-learner collaborations (Zainuddin, 2018). The flipped approach offers priority to learners where they are all involved in their learning and the educator turns into the “guide on the side” (Baker, 2000 as cited in Suo and Hou, 2017). Concerning the active learning in FC, one could argue that the classroom activities in such classes lead to a positive learning context, which increases students' levels of involvement and engagement (Jamaludin and Osman, 2014). Furthermore, engaging learners in problem-solving boosts their connections and confidence for their own educations, leading to higher emotional involvement (Lin, 2017, 2019), and at the same time, students' intellectual engagement increases when they have the option to ask questions that determines the influence of flipped classroom on student interaction (Schussler, 2009).

## Implications and Future Directions

The present review probes into the role of FC in translation teaching and its effect on students' engagement as well as the student and teacher interactions. The present review has noteworthy implications for teaching translation. Indeed, by implementing FC in translation classrooms, students are encouraged to interact more and improve communication between educators and learners (Cen, 2018). So, to encourage learners to participate, the teacher provides useful video lectures for learners in advance to make them for discussion (Zainuddin and Perera, 2019). The teacher should provide problem-solving tasks for the learners as these types of tasks aim to engage students in the class discussion and develop their accountability for learning and help students become autonomous and problem solvers (Huang and Napier, 2015) and the activities should be designed in a way to trigger teacher–student interaction in class leading to the cultivation of critical thinking and creativity (Munir et al., 2018). The interaction embedded in FC can bring about more confidence (Cakici and Oflaz, 2012; Xie and Derakhshan, 2021) and provides the opportunity for more practice that impacts their enthusiasm and engagement (Kim and Kim, 2014; Derakhshan, 2021). In addition, the syllabus designers should design class activities in a way that activates the teacher and student interaction since it was a policy for them to share knowledge with peers and their teacher that promote their engagement (Zainuddin and Attaran, 2016). As principle and practice should be closely associated, translation educators are supposed to apply FC in practices in the process of teaching and instruction to enhance students' engagement and interaction. In particular, in the time of pandemic worldwide, EFL teachers who teach translation courses should adapt to this new online teaching as well focus on EFL learners' adaptation to flipped learning style. As a result, future experimental studies can be conducted to assure the effect of FC.

## Author Contributions

The author confirms being the sole contributor of this work and has approved it for publication.

## Funding

This study was sponsored by Research of the Translation Course Reform of English Major against the Background of Constructing the Discourse System of China's Engineering Technology of Research and Practice Project of Teaching Reform of Higher Education of Hebei Province (Approval No. 2018GJJG231).

## Conflict of Interest

The author declares that the research was conducted in the absence of any commercial or financial relationships that could be construed as a potential conflict of interest.

## Publisher's Note

All claims expressed in this article are solely those of the authors and do not necessarily represent those of their affiliated organizations, or those of the publisher, the editors and the reviewers. Any product that may be evaluated in this article, or claim that may be made by its manufacturer, is not guaranteed or endorsed by the publisher.
